# Accelerating the Uptake and Timing of Antiretroviral Therapy Initiation in Sub-Saharan Africa: An Operations Research Agenda

**DOI:** 10.1371/journal.pmed.1002106

**Published:** 2016-08-09

**Authors:** Sydney Rosen, Matthew P. Fox, Bruce A. Larson, Papa Salif Sow, Peter D. Ehrenkranz, Francois Venter, Yukari C. Manabe, Jonathan Kaplan, Models for Accelerating Treatment Initiation (MATI) Technical Consultation

**Affiliations:** 1 Department of Global Health, Boston University School of Public Health, Boston, Massachusetts, United States of America; 2 Health Economics and Epidemiology Research Office, Department of Internal Medicine, School of Clinical Medicine, Faculty of Health Sciences, University of the Witwatersrand, Johannesburg, South Africa; 3 Department of Epidemiology, Boston University School of Public Health, Boston, Massachusetts, United States of America; 4 Bill & Melinda Gates Foundation, Seattle, Washington, United States of America; 5 Department of infectious Diseases, University of Dakar, Dakar, Senegal; 6 Wits Reproductive Health and HIV Institute, Department of Internal Medicine, School of Clinical Medicine, Faculty of Health Sciences, University of the Witwatersrand, Johannesburg, South Africa; 7 Division of Infectious Diseases, Department of Medicine, Johns Hopkins School of Medicine, Baltimore, Maryland, United States of America; 8 Independent investigator, Atlanta, Georgia, United States of America

## Abstract

Sydney Rosen and colleagues describe an operations research agenda to accelerating uptake of HIV treatment initiation.

**Summary Points**

- Under 2015 World Health Organization guidelines calling for antiretroviral therapy (ART) for all HIV-infected persons (the “treat all” approach), millions of new patients will be eligible to initiate ART, but existing procedures for treatment initiation are cumbersome and slow, contributing to high loss to follow-up before antiretroviral medications are dispensed. Simpler, more efficient, accelerated algorithms for ART initiation are needed.

- The Models for Accelerating Treatment Initiation (MATI) technical consultation developed an operations research agenda to build an evidence base for accelerating ART initiation. It focused on the operational question of how to start ART, with “how” encompassing timing and speed, required laboratory tests and the technologies for performing them, where to initiate (in the clinic, community, or home), the quantity and content of counseling and education needed, and the roles of different cadres of service providers, including facility- and community-based healthcare workers.
- Six priority research questions for optimizing the initiation process were identified. The highest priority is to evaluate whether a simplified clinical algorithm in patients who have tested positive for HIV can safely identify patients who should and should not start ART immediately without awaiting further laboratory test results, followed by determining the optimal speed of initiation and how to streamline clinic operations to make initiation more efficient.
- Proposed standardized outcomes to measure in research on models of treatment initiation include ART initiation within 28 days of first HIV-related clinic visit, six-month retention in care (clinic visit within 90 days of the expected six-month visit after initiation), and early viral suppression (suppressed viral load within 28 days of the expected first routine viral load test).

## Introduction

In 2015, the World Health Organization (WHO) recommended initiating lifelong antiretroviral therapy (ART) for all patients testing positive for HIV, regardless of CD4 cell count [1]. WHO cited three anticipated benefits from this “treat all” approach (also called “test and start” or “test and treat”): reduced morbidity among HIV-infected patients, reduced risk of transmission from HIV-infected individuals to their partners, and “increases in ART uptake and linkage to care, reduction in the time between HIV diagnosis and ART initiation regardless of baseline CD4 cell count and an increase in the median CD4 value at ART initiation.” A recent analysis suggests that future reductions in HIV-related mortality in sub-Saharan Africa will derive largely from this last benefit, rather than from the first two [2].

Although for budgetary and other practical reasons many low- and middle-income countries continue to apply a CD4 threshold to determine ART eligibility, it is clear that the trend is toward offering treatment to all those diagnosed with HIV. As this happens, the importance of “pre-ART care,” defined as care in the interval between HIV diagnosis and ART initiation, will diminish, along with the well-documented challenge of retaining patients in pre-ART care [3,4]. One of the challenges that will replace it is that of initiating newly diagnosed individuals on ART as efficiently as possible, while ensuring that patient autonomy, welfare, and retention on ART are not jeopardized by the initiation process.

Studies from throughout sub-Saharan African continue to document high losses of treatment-eligible patients from care before they receive their first dose of antiretrovirals (ARVs), due to a wide range of facility- and patient-level barriers to initiation [5]. Multiple required visits, long waiting times, stock outs of supplies, staff absences, and poor communication between staff and patients all deter treatment initiation [6–9]. Many of these barriers will remain under a treat-all policy that does not also solve the problem of linking patients to care or initiating them on ART efficiently. Patients tested in the community will continue to require referral to a facility providing ART. The need to screen for and treat tuberculosis (TB) [10] and cryptococcal infection [11,12] before starting ART will also remain. Retention of patients on ART in the months after treatment initiation, moreover, may depend in part on the manner of treatment initiation. Simpler, more efficient, and faster algorithms for ART initiation will be needed if the treat-all approach is to realize the benefits expected.

To begin to develop such algorithms, the Models for Accelerating Treatment Initiation (MATI) technical consultation was held in October 2015 (see S1 Text for the meeting agenda). The MATI consulation was premised on the observation that while many studies have evaluated what to start (regimens) and when to start (eligibility), few have tackled the operational question of how to start ART, with “how” encompassing issues of timing and speed, required laboratory tests and the technologies for performing them, where to initiate, the quantity and content of counseling and education needed, and the roles of different cadres of service providers.

## Research Agenda

The MATI consultation developed a list of priority operations research questions on how to optimize algorithms for treatment initiation in sub-Saharan Africa, with the goal of maximizing the number of patients who can be initiated on ART given available financial, infrastructural, and human resources and without jeopardizing treatment outcomes. Box 1 lists some of the topics that were addressed. Here we present the six research priorities that were identified and briefly discuss each one. We note that these priorities and the outcomes defined in the next section pertain to general adult populations. Pregnant and postpartum women, children and adolescents, and populations facing specific obstacles in accessing care (e.g., migrants, sex workers) may call for different approaches than those suggested here.

**Box 1. Characteristics of Treatment Initiation Addressed by the MATI Technical Consultation**

**Patient populations:** Who will be starting ART in the future?

- Many patients will be diagnosed with HIV for the first time
- Some patients will have been monitored in pre-ART care but were not previously eligible for ART
- Many patients will have been previously diagnosed and lost from care before starting ART
- An increasing number of patients will be re-initiators—patients who received ARVs at some time in the past but then stopped treatment

**Timing and speed of treatment initiation:** Once a patient has been diagnosed, how quickly can treatment be started without risking starting “too fast” and jeopardizing patient welfare or post-initiation outcomes and retention?

- Number of clinic visits required
- Time interval between visits and from start to finish

**Minimum clinical information required:** What do clinicians need to know before they prescribe ARVs, and what is the most efficient way to generate this information?

- Risk of TB
- Risk of cryptococcal meningitis
- Other immune reconstitution inflammatory syndrome (IRIS) risks
- Information from physical examination
- Results of blood tests to select ARV regimen (e.g., creatinine clearance)

**Minimum counseling and education required:** What are the optimal number, duration, timing, staff cadre, and content of nonclinical interactions to ensure that patients are able and willing to adhere to ART?

- HIV/ART/adherence education
- Individual and/or group counseling
- Optimal staff cadre providing services (nurses, counselors, other)
- Counseling before or after initiating medications

**Location of ART initiation:** Can ART be initiated successfully in nonclinical locations?

- HIV testing and ART initiation on-site (at a clinic)
- HIV testing off-site and referral to a clinic for initiation
- HIV testing and initiation off-site (e.g., home-based or other community locations)
- Role of community volunteers (e.g., community health workers) and clinic-based professional staff in supporting initiation in community and/or clinic settings
- Effect of location of initiation on early retention on ART

**Patient behavior and decision-making:** How can acceptability of ART initiation be improved?

- Known and new patient barriers to enrollment and initiation
- Effect of model of ART initiation on patient uptake of ART (will more patients accept ART if initiation is easier?)
- Importance of targeting initiation models to different patient populations
- Care for patients who choose not to start ART (either not now or not ever)

**Supply side:** What do health systems need to have and do for accelerated initiation?

- Role of health system context in choosing model(s) of treatment initiation
- Reducing known barriers to initiation created by healthcare providers, such as long waiting times and patient record systems that inhibit effective follow-up
- Infrastructural, procurement, human resource, and other provider requirements and bottlenecks for accelerated initiation

**Demand side:** How many more patients will seek ART initiation?

- Pace of adoption of “test and start” at the country level
- Proportion of HIV-infected populations that will remain undiagnosed or decline treatment
- Capacity of existing models and/or need for new model(s)

**Measuring success and data requirements:** How should we evaluate different models of ART initiation?

- Outcomes (definitions for ART initiation, viral suppression, early retention on ART)
- Provider costs and cost-effectiveness
- Patient costs and benefits

### Question 1: What does it take to start ART under current practices, and how will this change when the 2015 WHO guidelines are adopted, as measured by number of clinic visits needed, duration of process from start to end, laboratory tests and other clinical procedures required, counseling and education required, and resources utilized?

Question 1 asks for a baseline of actual practice to provide a better understanding of how treatment initiation is currently being conducted. There is very little published evidence on the practical details of the process and the extent to which it varies by facility, setting, or country. Without a robust baseline evidence base, it is challenging to identify opportunities for making improvements.

Answering question 1 in a comprehensive way will require data collection in multiple countries and settings. Fortunately, most of the data required can be generated relatively quickly, by administering detailed cross-sectional questionnaires on procedures and resources to facility-level staff. A medical record review to estimate actual numbers of clinic visits, services provided, and duration from start to finish will also be needed in most settings.

### Question 2: Can a simplified clinical algorithm comprising a symptom report, medical history, readiness assessment, and brief physical exam in HIV-positive patients reliably identify patients who should and should not start ART immediately (during the same clinic visit), and will such an algorithm increase the proportion of patients who initiate ART and achieve viral suppression, compared to standard care?

**Fig 1:**
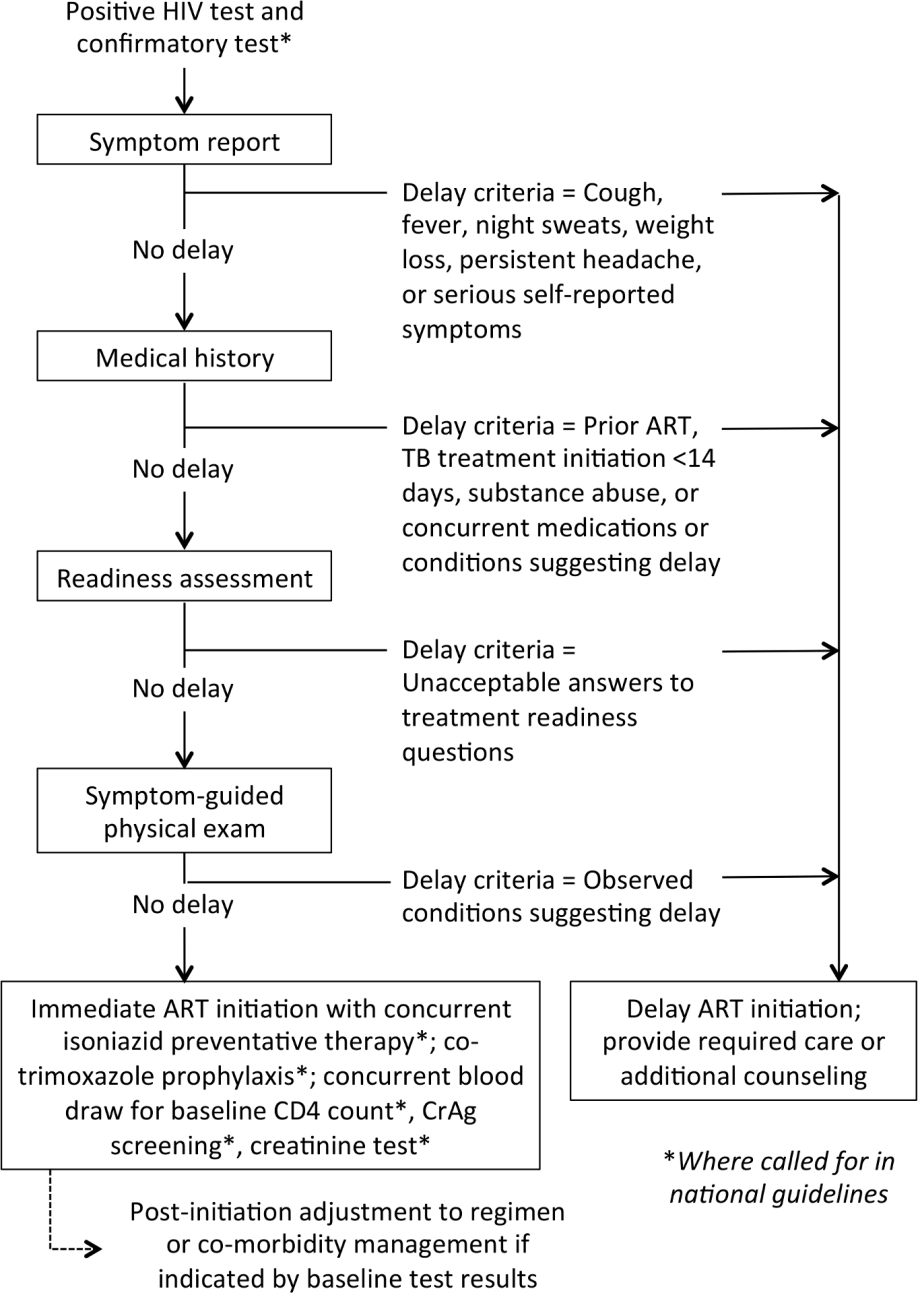
Determining clinical eligibility for immediate ART initiation: proposed algorithm for evaluation in the research agenda. Diagram showing the steps that could be included in an algorithm to evaluate whether HIV-positive patients are eligible for immediate ART initiation or instead require additional care or services before medications are dispensed. The MATI consultation recommended that this algorithm be evaluated in research studies. CrAg, cryptococcal antigen.

Question 2 was considered the highest priority research question by MATI consultation participants. The diagram in Fig 1 illustrates the new algorithm that the MATI consultation recommended should be evaluated. It is premised on the expectation that once there is no longer a CD4 count threshold in place, a large proportion of those who test HIV-positive will be clinically eligible for immediate initiation, and that we should not delay starting treatment for the majority of patients who are ready to start immediately in order to prevent starting too quickly for the minority who are not. Question 2 posits that an algorithm based on a simple symptom report, medical history, treatment readiness assessment, and limited physical exam in patients who have tested positive for HIV will identify patients who should and should not start ART immediately with sufficient accuracy to improve overall health outcomes. The algorithm is based on the hypothesis that the benefits gained from immediate initiation, in terms of reduced loss to follow-up, exceed any risks from adverse responses to ART that may also result from immediate initiation.

Under the algorithm, HIV-positive patients who have acceptable results for the symptom report, medical history, readiness assessment, and physical exam can be deemed clinically eligible to be dispensed ARVs on the same day, without waiting for other laboratory test results or other steps. Baseline tests such as CD4 count, creatinine, and/or other blood tests, and cryptococcal antigen (CrAg) screening where indicated, could still be done but would not delay ART initiation. Nonclinical steps, such as counseling and education about ART adherence, could be done on the same day and/or at follow-up visits, without losing the value of these activities.

Initiating ART in patients with underlying conditions such as TB or cryptococcal infection who were not screened out by the simplified algorithm would increase the risk of IRIS or death. For TB, the algorithm symptom screen includes the standard TB symptoms already used to identify TB suspects in most settings, such that the risk of TB IRIS under the new algorithm would likely not change relative to current practice. WHO guidelines recommend isoniazid preventive therapy [13] to reduce TB mortality risks in asymptomatic HIV patients with low CD4 counts [14,15] and co-trimoxazole prophylaxis for patients with CD4 counts below 350 cells/μl [16]; both isoniazid preventive therapy and co-trimoxazole prophylaxis could augment the simplified algorithm. Cryptococcal infection is more challenging; in countries with routine CrAg screening, a patient with a positive CrAg test would need to be contacted promptly and receive appropriate care.

Whether the benefits of the simplified algorithm exceed the risks is a question that should be evaluated empirically. The research to answer question 2 will likely entail randomized trials to generate an evidence base that represents a range of settings and populations. While such trials will take some time to launch, if high-volume study sites are chosen, enrollment and follow-up can be completed quickly, as outcomes (uptake of ART and retention and attrition in the first six months after initiation) can be assessed very soon after study enrollment. Evaluations of programs that offer immediate ART to HIV-positive pregnant women [17,18] may also provide clues about how a simplified algorithm like that in Fig 1 will perform in the general adult population.

### Question 3: What is the optimal timing and speed of initiation, including the number of clinic visits and the duration of the process from start to finish, as measured by early ART outcomes and cost-effectiveness?

Question 3 addresses how fast is “fast enough” or “too fast” for the ART initiation process. Options include single-visit initiation (also called “same day” initiation), in which ARVs are dispensed during a patient’s first HIV-related clinic visit, which might occur on the same day as HIV diagnosis; a two-visit process that allows time for laboratory tests and personal acceptance of the need for treatment in between the two visits; or an initiation process that includes more than two visits, including the status quo, which typically requires anywhere from three to six visits. The optimal number of days between visits is also relevant to question 3: a three-visit process in which all visits are completed in one week may have different consequences for patients than the same number of visits spread over six or eight weeks.

Two new randomized trials [19,20] and a single-arm program evaluation [21], while taking very different approaches, all found that accelerating the process of ART initiation resulted in very high uptake of ART and, in the case of the trials, significantly better health outcomes for patients. Other relevant studies are underway, and it is likely that a wide range of innovations aimed at speeding up treatment initiation are being implemented in nonresearch contexts. If these experiments are evaluated properly, they may also contribute to the evidence base. A challenge in considering alternative approaches is heterogeneity in the starting point for evaluation: some studies begin with the HIV test, other studies count only clinic visits made once a patient has enrolled in care, and still others start only when treatment eligibility has been determined.

Additional questions related to speed and timing include the following: (1) Is more than one adherence education session needed to generate good patient outcomes, or is one session enough? (2) Do patients themselves prefer having a single visit or multiple visits? (3) Does the speed or timing of visits prior to treatment initiation affect outcomes after initiation?

These questions are likely to have different answers for different patient populations, health systems, and settings. The pace of guideline changes and the many programmatic innovations already underway may overtake any randomized controlled trials that are launched now, arguing for investment in rigorous evaluations of initiatives already underway, rather than the development of new trials.

### Question 4: What changes to clinic management, capacity, and resources are needed to support accelerated ART initiation, and particularly same-day initiation?

Once effective approaches to accelerating treatment initiation are identified, adjustments to health clinic practices and capacity will be needed to implement them in routine practice and at scale. Changes may be necessary in staff training, responsibilities, schedules, and performance management; patient-to-provider ratios; clinic space allocation; the flow of patients through the clinic; inventory storage and tracking; and data management. The studies of accelerated initiation cited above all provide some clues as to what kinds of changes may be needed. Resource constraints—limits in staff time and motivation, consultation rooms, patient waiting areas, and even stationery for record keeping—will be a concern in many settings. Any effort to accelerate or improve ART initiation is likely to require better data management than is currently in place in most clinics, so that patients can be tracked accurately and promptly, requiring investments in data systems as well as human and infrastructural resources.

Question 4 is an operational question that will likely be answered through an iterative empirical approach as governments and treatment support programs adjust to new recommendations on accelerated treatment initiation and absorb the larger numbers of eligible patients under the new WHO guidelines. From a research perspective, much of this work will fall within the domain of those who evaluate health system performance or undertake quality improvement assessment, with quantified patient outcomes as the desired endpoints.

### Question 5: Is initiation of ART outside of clinics (community- or home-based ART initiation) safe, effective, and cost-effective?

Moving HIV-related service delivery from clinics to other locations is a major goal of WHO recommendations and is the focus of a great deal of recent research. While there is a long history of community-based HIV testing, and increasing evidence about managing stable ART patients outside the clinic, the actual initiation of ART in nonclinical settings or in patients’ homes is a relatively new topic [22].

The potential benefits of initiating treatment in community- or home-based settings include increased access, reduced patient costs, reduced crowding in clinics, and better uptake of ART. The potential costs, or harms, include poorer retention in care after initiation for patients who never get “established” in an ART program, more adverse events related to the lack of clinical attention to the patient, and the program costs of service delivery in other locations. Patient density, distances to facilities, and the health worker cadre employed to provide the services will have a large effect on costs. It is unclear whether existing human resources are sufficient in numbers and training to supervise home-based initiation, even if there is reduced patient volume at clinic facilities. It is also important that data systems reliably capture non-clinic-based services, so that patients can continue to be monitored by clinical providers.

Research in a wide range of countries and populations, using different service delivery approaches, will be needed to determine whether the benefits exceed the costs and are sustainable outside research settings. Studies are currently being launched [23], and there are likely to be numerous opportunities to evaluate programs already underway. A mathematical cost-effectiveness model could be developed that would help identify the conditions under which community- or home-based service delivery programs are likely to be cost-effective and affordable.

### Question 6: Why do some patients not start ART when advised, and which interventions will be effective in changing behavior to increase and accelerate ART uptake?

The first five questions on the research agenda address the supply side of treatment initiation—how to make the service offered more accessible and efficient. Question 6 turns to the demand side. No matter what is offered by providers, some patients will not start treatment despite being eligible. For those who are never diagnosed or never reach the point of being advised of their eligibility for treatment—populations not addressed in this paper—more effective interventions for HIV testing and linkage to care will be needed [24]. For those who are advised to initiate but decline to do so or delay the process indefinitely, research is needed to understand their reasoning and encourage them to reconsider. It is unclear whether and how different models of treatment initiation affect demand for ART uptake. Better, more patient-friendly approaches may overcome nonclinical barriers to uptake, or the reasons that eligible patients do not start treatment may have little to do with real or perceived service delivery barriers [25]. Actual rates of treatment refusal and the duration of delays between eligibility and initiation are largely unknown, both because of the difficulty of studying what patients do *not* do and because few health systems are able to trace patients between facilities or even over time. As with much research on the HIV cascade of care, a universal patient identifier that allows tracing of patients would greatly benefit research on how to accelerate ART initiation.

### Research Outcomes for Evaluations of ART Initiation

A major obstacle to interpreting and synthesizing existing research on ART initiation is the heterogeneity in outcomes reported. The MATI consultation participants thus also proposed a standard set of primary and secondary outcomes for studies aimed at accelerating or increasing uptake of ART initiation. While the focus of these studies is ART uptake, early outcomes on treatment were also considered important and may be affected by the model of treatment initiation. Whenever possible, studies of ART initiation should start with HIV diagnosis and follow patients through a minimum of six months after treatment initiation (or the point at which treatment should have been initiated). Intermediate endpoints, such as linkage to care or determination of treatment eligibility, remain important, but on their own are not sufficient to evaluate the effectiveness of a new model of service delivery. Proposed primary outcomes are described in detail in Table 1. Secondary outcomes that were identified as potentially important include cost, acceptability, scalability, and generalizability (Table 2).

**Table 1:** Proposed primary outcomes for evaluation of ART initiation in general adult populations.

Outcome	Parameter	Definition
**ART initiation within 28 days**	Equation	ART initiation equals the number of treatment-eligible patients initiating ART within 28 days of first HIV-related clinic visit divided by the total number of treatment-eligible patients.
	Timing	The interval allowed for patients to initiate ART after clinic presentation varies in previous reports, with recent studies using intervals ranging from 14 days [20] to 90 days [19]. In all the studies presented at the MATI consultation, more than half of patients in the standard of care arm initiated treatment within one month (28 days) of initial clinic presentation, confirming that one month is sufficient time for all procedures to be completed. Within 28 days of first treatment-eligible, HIV-related clinic visit was thus recommended as the standard interval for this outcome. We also note that the starting point of the interval is the first interaction with the healthcare system at which the patient is eligible for ART. Under the new WHO guidelines, this will coincide with a positive HIV test, as diagnosis and eligibility will be simultaneous. Until the treat-all approach is adopted, it is important to specify the starting point.
	Denominator	All patients found to be eligible for ART during the study enrollment period (study cohort). Although nearly all patients will be eligible once the treat-all approach is adopted, for the time being most countries continue to apply a CD4 count threshold for eligibility. The denominator for this outcome should include all patients who are eligible, whether or not their eligibility has been conveyed to them. Thus, a patient who has a CD4 count under the threshold but does not return to obtain the CD4 count result should be included in the denominator. The enrollment period should be specified with starting and ending dates.
	Numerator	Patients in the study cohort who are dispensed their first supply of ARV medications within the allowed interval (28 days). Where prescribing and dispensing are separate steps, dispensing to the patient is the preferred indicator.
Six-month retention in care*	Equation	Early retention in care equals the number of patients retained in care six months after the expected date of treatment initiation divided by the number of treatment-eligible patients expected to have initiated ART.
	Timing	Since the reason for post-initiation follow-up in studies like those proposed here is to ensure that the manner of ART initiation does not harm post-initiation outcomes, short-term retention in care is a reasonable and readily measurable outcome. Six months from the expected date of treatment initiation is recommended as the standard interval for this outcome.
	Denominator	All patients found to be eligible for ART during the study enrollment period (study cohort). Because we are evaluating treatment initiation, not retention in care, a patient who is eligible for treatment but never initiates should be included in the denominator. For this outcome, there is no ART initiation date for patients who are lost before initiation. For these patients, the outcome could be assessed one month (28 days) plus the specified interval (e.g., six months) after the first HIV-related clinic visit. This allows the patient the same 28 days to start ART as suggested for the ART initiation outcome, plus the same duration of potential follow-up as the patients who did start ART. The enrollment period should be specified with starting and ending dates.
	Numerator	Patients in the study cohort who fulfill the selected definition of retained in care, which is typically not more than a specified number of days late for the next scheduled visit. Definitions of “retained” will vary by national guidelines, data availability, and researcher norms; not more than 90 days late for the next scheduled medication pickup is a commonly used definition. This outcome can be used even when viral load tests are not done, as it depends only on clinic visit data.
**Early viral suppression**	Equation	Early viral suppression equals the number of patients virally suppressed at first routine viral load test divided by the number of treatment-eligible patients expected to be virally suppressed.
	Timing	The timing of this outcome will depend on when routine viral load tests are done under national guidelines in countries that adopt viral load monitoring. WHO recommends the first routine viral load test at six months after treatment initiation [26], but many countries wait until 12 months. For countries with the first routine viral load test at 12 months, viral suppression by 13 months after the expected date of treatment initiation could be used as the outcome, allowing patients a one-month window after the scheduled date while also capturing viral loads that may have been suppressed before the 12-month point. For countries with the first routine viral load test at six months, viral suppression by seven months after the expected date of treatment initiation could be used as the outcome.
	Denominator	All patients found to be eligible for ART during the study enrollment period (study cohort). Because we are evaluating treatment initiation, not retention in care, a patient who is eligible for treatment but never initiates should be included in the denominator and considered not to have reached the outcome. For this outcome, there is no ART initiation date for patients who are lost before initiation. For these patients, the outcome could be assessed one month (28 days) plus the specified interval (e.g., 12 months) after the first HIV-related clinic visit. This allows the patient the same 28 days to start ART as suggested for the ART initiation outcome, plus the same duration of potential follow-up as the patients who did start ART. The enrollment period should be specified with starting and ending dates.
	Numerator	Patients in the study cohort who have a recorded viral load below the definition threshold for suppression that is used in the country. There is variation in this threshold, and using different countries’ standards may reduce the comparability of studies. For operations research, however, it is appropriate to use the accepted local threshold (and this is likely what routinely collected data will permit, as well). Patients who do not have viral load test results recorded should not be included in the numerator for this outcome.

*For all the outcomes in Table 1, but particularly for retention in care, patients who transfer to other clinics before reaching the outcome endpoint pose an analytic challenge, as their outcomes are usually unknown. For patients who transfer formally, it may be possible to obtain the outcome from the new clinic, or the transfer may occur close enough to an endpoint to infer it from available data. Patients who self-transfer without informing the original clinic (so called “silent transfers”) are typically counted as not retained, a practice that may overestimate actual loss to care but is difficult to correct without active tracing of defaulters or a universal patient identifier.

**Table 2:** Proposed secondary outcomes for evaluation of ART initiation in general adult populations.

Outcome	Description
Cost, affordability, and cost-effectiveness	In situations of constrained budgets, knowing how much a different model of ART initiation will cost to implement, compared to current costs, is essential for governments and funding agencies. Estimating cost-effectiveness compared to the status quo or to alternative models is also critical. Most models for accelerating ART initiation will have different costs of service delivery than standard care; whether these costs are justified will depend on how effective the model is in achieving better outcomes. In most settings, provided that the primary outcomes are measured consistently, there is no immediate programmatic need to estimate utility outcomes such as cost per quality-adjusted life year or cost per disability-adjusted life year. These outcomes can be modeled secondarily when needed. Because accelerated models of ART initiation are also likely to provide benefits to patients—fewer clinic visits, less time spent waiting at the clinic—the benefits and costs of the models to patients should be included in the economic evaluation whenever possible.
Acceptability	No matter how technically efficient a model of service delivery is, it will fail if patients or communities do not find it acceptable. For operations research studies, uptake of the intervention (equal to one minus the refusal rate) is easy to measure and could be considered as a proxy indicator of acceptability, though it does not provide reasons for choices. Supplemental qualitative research to understand patients’ preferences for different models of service delivery should be included in studies whenever possible.
Scalability	There is no commonly used definition of scalability. In many publications, the term is used interchangeably with feasibility: if the researchers could do it in their study, it must be scalable. A practical definition may incorporate the incremental resource requirements for providing a particular model of treatment initiation to a population. For example, for every 1,000 HIV-positive adults not yet on ART, how many *additional* nurses, counselors, laboratory tests, computers, training sessions, clinic rooms, and other resources will be required to implement the selected model, and which of these resources will constitute bottlenecks? While this analysis is related to cost, it aims to position the intervention within the context of an existing program and thus to gauge how realistic it is to propose large-scale adoption of the intervention.
Generalizability	Many publications dismiss the specific characteristics of the patients enrolled in a study as a study limitation, rather than actively addressing the question of which populations the results apply to. For new models of service delivery, determination of the relevant population is a critical step in deciding whether and how to implement the model beyond the study sites. Including in a study an explicit analysis of generalizability can thus greatly assist policy makers in choosing whether to invest in scale-up.

## Conclusion

In each country that adopts the treat-all approach to HIV care, large numbers of new patients will become eligible for ART initiation. Not all of them will volunteer for HIV testing, and many who already know their status will decline to enroll in HIV care. Despite this, we should expect to offer ART initiation to hundreds of thousands, perhaps millions, of new patients, including both newly diagnosed patients and those who previously sought care but were not eligible for ART. Initiating all of these additional patients on treatment without incurring high losses to follow-up will require new approaches to the “how” of treatment initiation. The research agenda proposed here aims to identify and evaluate such approaches, so that countries can adopt appropriate strategies that are backed by evidence of effectiveness and cost-effectiveness and make optimal use of available resources.

## Supporting Information

S1 Text Agenda for the MATI technical consultation. (PDF)

## Abbreviations

ART: antiretroviral therapy
ARV: antiretroviral
CrAg: cryptococcal antigen
IRIS: immune reconstitution inflammatory syndrome
MATI: Models for Accelerating Treatment Initiation
TB: tuberculosis
WHO: World Health Organization
