# Supplementary material for: Accelerating the Uptake and Timing of Antiretroviral Therapy Initiation in Sub-Saharan Africa: An Operations Research Agenda
Source: PLoS Med. 2016 Aug 9;13(8):e1002106. doi: 10.1371/journal.pmed.1002106 (PMC4978457; doi:10.1371/journal.pmed.1002106)
Supplement: S1 Text — (PDF) [file pmed.1002106.s001.pdf]

Models for Accelerating Treatment Initiation
Technical Consultation Agenda

Day 1 (October 21, 2015)—Get the Information on the Table

Time	Session	Speakers
8:30-9:00	Registration	
9:00-9:30	Introduction/Objectives/Agenda	Papa Salif Sow, Sydney Rosen
**Session 1: Data (Session Chair: Papa Salif Sow)**		
09:30-9:50	*Existing Data*	
	Review of Published Studies on Models of ART Initiation	Matthew Fox
9:50-10:45	*New Data (10 minute presentations)*	
	RapIT: Initiating ART on the First Visit	Sydney Rosen
	Point of Care Laboratory for ART Initiation	Lesley Scott
	START: Streamlined ART Start Strategy	Charles Holmes on behalf of Elvin Geng
	Fast-Track ART Initiation	Lynne Wilkinson
	Home-Based Linkage and ART Initiation	Ruanne Barnabas
10:45-11:00	Break	
11:00-11:45	Questions and Discussion of Data Presented	Panel: All Presenters from Session 1; Nathan Ford (Facilitator)
11:45-12:15	General discussion	All Participants
12:15-01:15	Lunch	
**Time**	**Session**	**Speakers**
**Session 2: Issues to Consider (Session Chair: Sydney Rosen)**		
1:15-2:15	*Clinical Issues (10 minute presentations)*	
	Tuberculosis Diagnosis and Treatment and ART Initiation	Yuka Manabe
	Cryptococcal Meningitis Screening and Treatment and ART Initiation	Bruce Larson
	Clinic Capacity and Constraints for ART Initiation	Francois Venter
	Community Capacity and Constraints for ART Initiation	Morten Skovdal
	Care for Eligible Patients Who Decline ART	Ribakare Muhayimpundu
2:15-2:45	Questions and Discussion of Topics Presented	Panel: All Presenters on Clinical Issues
2:45-3:00	Break	
3:00-3:30	*Technical Issues (10 minute presentations)*	
	Cost and Cost-Effectiveness Considerations	Paul Revill
	Data Systems for Accelerating ART Initiation	Meg Osler
	Role of Laboratories in ART Initiation	John Nkengasong
3:30-4:00	Questions and Discussion of Topics Presented	Panel: All Presenters on Technical Issues
4:00-4:15	What Information Do National Governments Need?	Yogan Pillay
4:15-4:45	International Technical Agency Approaches (WHO and IAS)	Nathan Ford, Anna Grimsrud

4:45-5:00	General Discussion and Wrap-Up of Day	All Participants
6:00 onwards	Gathering at Dopio Zero, Corner Church St and St. George's Mall	All Welcome

Day 2 (October 22, 2015)—Discuss What to Do About It

Time	Session	Speakers
09:00-09:15	Introduction/Objectives/Agenda	Sydney Rosen
**Session 1: International Agency and Government Perspectives (Session Chair: Matt Fox)**		
9:15-10:15	Panel Discussion of Donor Priorities and Plans: PEPFAR (CDC, USAID), Global Fund, Bill & Melinda Gates Foundation	Jon Kaplan, Annette Reinisch, Peter Ehrenkranz, Carol Langley
10:15-10:30	Break	
**Session 2: Review of the Evidence Base (Session Chair: Peter Ehrenkranz)**		
10:30-11:30	What Do We Know? Inventory of Delivery Models and Data	Tendani Gaolathe, Charles Holmes (Facilitators)
11:30-1:00	Breakout Group Discussions: What Do We Need to Know? Gaps in the Evidence Base and Opportunities to Fill Them	Breakout Groups, Topics TBD
1:00-2:00	Lunch	
2:00-2:40	Report Back and Full Group Discussion: What Do We Need to Know? Gaps in the Evidence Base and Opportunities to Fill Them	Francois Venter (Facilitator)
**Session 3: Research Agenda (Session Chair: Papa Salif Sow)**		
2:40-3:30	Data Quality, Designs, and Key Outcomes for Evaluation	Matt Fox (Facilitator)
3:30-3:45	Break	
3:45-4:15	Prioritization of Research Questions	Ruanne Barnabas (Facilitator)
4:15-4:30	Wrap-Up and Next Steps	Papa Salif Sow, Sydney Rosen
